# Diagnostic radiographers’ perspectives on dental radiography in selected provinces in South Africa

**DOI:** 10.4102/hsag.v30i0.3043

**Published:** 2025-07-25

**Authors:** Keshini Govindasami, Shenuka Singh

**Affiliations:** 1 Discipline of Public Health, School of Nursing and Public Health, University of KwaZulu-Natal, Durban, South Africa; 2 Discipline of Dentistry, School of Health Sciences, University of KwaZulu-Natal, Durban, South Africa

**Keywords:** diagnostic radiographer, dental radiography, perspectives, knowledge, practice

## Abstract

**Background:**

Dental radiography forms part of the scope and training for a diagnostic radiographer in South Africa; yet there is limited published evidence related to these professionals’ knowledge, attitudes and practices on dental radiography.

**Aim:**

This study aimed to determine the knowledge, attitudes and practices of diagnostic radiographers on dental radiography.

**Setting:**

This study was conducted in three South African provinces, namely, KwaZulu-Natal, Gauteng and the Western Cape.

**Methods:**

This study used a cross-sectional, correlational design, using purposive and snowball sampling techniques. An online questionnaire was used for data collection, and the data were analysed using SPSS version 29.0.

**Results:**

A study response rate of *N* = 207 was obtained, where 75.4% of participants agreed that they were aware of what dental radiography entails, 68.1% of participants reported not having adequate knowledge of the necessary dental radiography techniques and 72.4% of participants did not know the key anatomical positioning landmarks in dental radiography. Almost 68.6% of participants disagreed with being able to practice dental radiography techniques confidently and independently. About 87.5% of participants agreed that they are willing and keen to learn about dental radiography, and 89.9% of participants were open to gaining clinical experience in dental radiography.

**Conclusion:**

There were inconsistencies in participants’ dental-specific knowledge and practical skills. There was a positive attitude among participants who showed a willingness to learn dental radiography.

**Contribution:**

This study reiterates the value of investing in continuous professional development activities to address identified knowledge, attitude and practice gaps among diagnostic radiographers in dental radiography.

## Introduction

Radiography is largely a skills-based profession, and the need for radiographers’ formal knowledge to be supplemented with exposure to ongoing practical and technical skills is critical (McNulty, England & Shanahan 2021). Several studies conducted on radiography education and training have depicted that when formal knowledge is not adequately supplemented with appropriate exposure to practical and technical skills, then this affects image quality that in turn has a detrimental effect to patient diagnosis (McNulty et al. 2021; Sa Dos Reis et al. 2018; Van de Venter and Engel-Hills 2022). There is thus a need for educational curricula to be focused on developing clinically competent healthcare professionals with appropriate knowledge and technical and practical skills to navigate within their professional scope in a wide range of healthcare settings (Sa Dos Reis et al. 2018; Van de Venter and Engel-Hills 2022). Among such healthcare settings is the field of dental radiography, where diagnostic radiographers are expected to work.

Dental radiography is crucial to the field of dentistry, as it provides essential diagnostic information to diagnose several orofacial anomalies, injuries and pathologies. It further helps in determining treatment options and aids in monitoring the success of the treatment (Khani et al. 2017; Rai et al. 2018). As dentistry is now directed more towards prevention than cure, the need for proper utilisation of advanced diagnostic modalities is essential for the detection of oral diseases prior to their physical manifestation (Thakkar et al. 2019). Therefore, all professionals working with dental radiography need to have a specific level of knowledge and technical and practical skills (De-Azevedo-Vaz et al. 2013; Thakkar et al. 2019).

An understanding of dental medical terminology, selecting the correct facial landmarks for concise positioning, appropriate use of intraoral assistive devices (e.g. intraoral film holders) and applying correct exposure and radiation safety are among some of the most relevant factors to consider when producing a dental image (De-Azevedo-Vaz et al. 2013; Rai et al. 2018). Several studies focusing on dental radiography have shown that if the above factors are not well executed, then this will result in inadequate dental image quality, which in turn results in poor oral diagnosis and has implications of increased radiation risk factors to patients and the surrounding environment (De-Azevedo-Vaz et al. 2013; Rai et al. 2018; Thakkar et al. 2019).

Several studies also iterate the need to develop innovative dental radiography education systems for diagnostic radiographers (Chen et al. 2022; Mahasneh et al. 2022). This would equip diagnostic radiographers with expert knowledge and technical and practical skills that would be required for producing high-quality dental images. Therefore, one such area that could contribute to an effective oral health system is understanding the practice and training of diagnostic radiographers in dental radiography. Dental radiography is within the scope of practice and training for South African (SA) diagnostic radiographers (Health Professions Council of South Africa [HPCSA] 2020), yet there is limited published evidence on the diagnostic radiographer’s attitudes towards practising dental imaging or their knowledge and skills to perform such tasks. Currently in SA, there are no studies covered in the field of dental radiography for diagnostic radiographers, and there is no evidence to show if diagnostic radiographers have the skills to effectively and efficiently contribute to dental radiography and thereby contribute to the oral healthcare needs of citizens in SA.

This study aimed to determine the knowledge, attitudes and practices of diagnostic radiographers on dental radiography.

## Research methods and design

### Study design

This study employed a cross-sectional correlational design to assess the knowledge, attitudes and practices of diagnostic radiographers regarding dental radiography and to examine the interrelationships among these variables using both bivariate and multivariate techniques (De Vaus 2001; Rajendra 2024).

### Target population and sample

**FIGURE 1:**
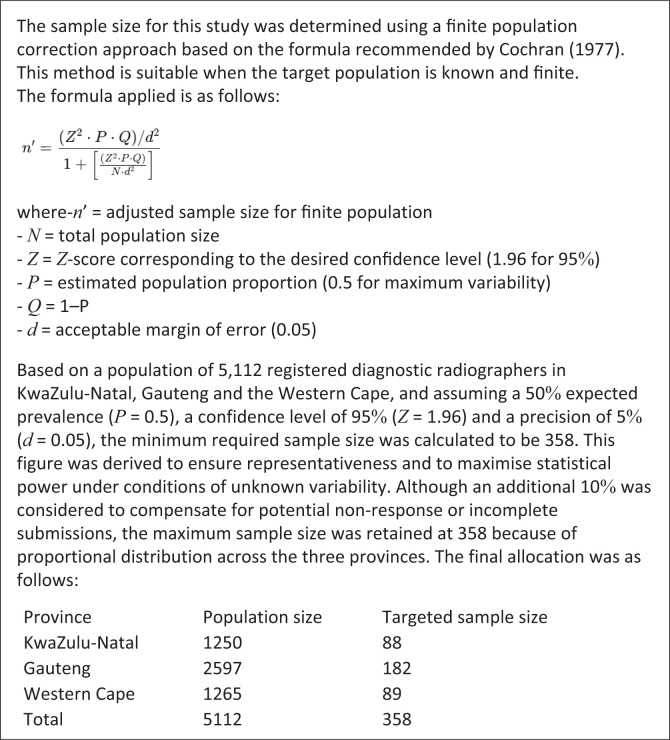
Statistical sample size formula.

Purposive sampling technique was utilised to sample qualified and community service diagnostic radiographers from both the public and private sectors in the provinces of KwaZulu-Natal, Gauteng and Western Cape (Polit & Beck 2010). The snowball sampling technique was also used to invite more study participants, where study participants were requested to distribute the study information to other potential individuals who may be interested in participating in the study (Polit & Beck 2010). According to the HPCSA register records in 2023, there were a total of 5112 diagnostic radiographers registered in the provinces of KwaZulu-Natal, Gauteng and Western Cape (Daffue 2023). This therefore allowed the researcher to sample from a larger number of diagnostic radiographers. Additionally, these provinces have many health institutions that offer oral health-care diagnostic services (Bhayat & Chikte 2018); hence, diagnostic radiographers working in these provinces would be more likely to have being exposed to dental radiography and, therefore, provided a greater wealth of information to answer study objectives. A statistician was consulted for the required sample size and data analysis. Cochran’s sample size formula for a finite population (Figure 1) (Cochran 1977) was used to calculate the targeted sample size (*n* = 358). (Figure 1).

### Participant recruitment

The study participants were recruited through social media platforms by the group administrators who posted study information on platforms such as WhatsApp and Facebook. Radiographers use these platforms to share information and communicate with each other on a professional basis. These groups are access controlled and are managed by the site administrators (Bhuiyan 2023a, 2023b; Jacobs 2023). The researcher forwarded the study information letter to these administrators who acted as gatekeepers. They reviewed the information before posting to the identified group.

In addition, the HPCSA referred the researcher to a survey distribution channel that the researcher could use to distribute the study information (Med Pages 1996). This survey distribution was managed by the site administrators, who facilitated access to this website. The study information was forwarded to the website administrators, who acted as gatekeepers. The website administrators reviewed the information prior to distributing the study information to diagnostic radiographers from selected provinces who were registered on their database. All interested participants were also requested to distribute the study information letter to other participants who may also be interested in participating in the study as a means of snowball sampling.

### Research instrument and data collection procedure

This cross-sectional survey was conducted between November 2023 and June 2024, after obtaining (ethical no: HSSREC/00005832/2023). The study involved an online self-administered questionnaire, which was used to determine qualified diagnostic radiographers’ knowledge, attitudes and practice as related to dental radiography.

The questionnaire was developed and adapted from previous knowledge, attitudes and practices studies conducted in dental radiography for dental professionals (Lavanya et al. 2016; Samejo et al. 2021). In addition, the questionnaire focused on questions found in literature on dental radiography (Manson-Hing 1990; Whaites & Drage 2013). Examples of these questions posed in the studies mentioned above were, ‘Do you have a panoramic radiography device in your clinic? Which technique do you utilize in taking periapical radiographs?’ (Lavanya et al. 2016; Samejo et al. 2021).

The data collection tool comprised four main sections, namely Section 1, which was based on the demographic details of the participants, including biological sex, age, year of experience and highest level of qualifications. Section 2 was based on participants’ knowledge of dental radiography, and Section 3 was based on attitudes of participants towards dental radiography and Section 4 on the practice that diagnostic radiographers have with respect to dental radiography. The study used a Likert scale format with responses ranging from 1 (strongly disagree), 2 (disagree), 3 (neutral) and 4 (agree) to 5 (strongly agree). The online questionnaire was developed, such that participants were required to select an option as related to the above scoring pattern prior to moving on to the next question; hence, all participants would have answered all questions.

The reliability analysis of the questionnaire was assessed using Cronbach’s alpha, demonstrating generally high internal consistency across most sections. The Knowledge, Attitudes and Practice (KAP) sections showed Cronbach’s alpha values of 0.831, 0.826 and 0.858, respectively.

### Data analysis

The questionnaire was the primary data collection tool, and the responses were analysed using SPSS version 29.0. Descriptive statistics, including measures of central tendency, variability and frequency, were used to summarise the data, which are presented in tables and figures. Inferential statistical analyses included correlation and Chi-square goodness of fit tests. The Chi-square (χ^2^) statistic is employed to assess the fit between observed and expected distributions, with *p*-values used to determine statistical significance (Balakrishan, Voinov & Nikulin 2013). A threshold of *p* < 0.05 was applied to identify statistically significant deviations, indicating non-random response tendencies (Balakrishan et al. 2013).

### Ethical considerations

Ethical clearance to conduct this study was obtained from the University of KwaZulu-Natal, Humanities and Social Sciences Research Ethics Committee (reference no: HSSREC/00005832/2023).

The online questionnaire link was developed on Google Forms and enclosed within the study information letter. Participants who were interested in participating after reading the study information letter accessed the questionnaire through the online questionnaire link. Prior to being able to access the online questionnaire link, participants were requested to first agree to the informed consent by clicking on the Agree icon, as this was taken as their informed consent to partake in this study. The online questionnaire link did not capture personal details of the participants and was set so that it did not capture email addresses of participants. Thus, participation was voluntary, and individual rights, interests, privacy and anonymity were upheld.

## Results

A total of 207 participants responded to the questionnaires, giving a response rate of 57.8% (*N* = 207). The gender disparity (*p* < 0.001) indicated that the gender distribution was statistically significantly different. Most participants were female, making up nearly three-quarters, that is 72.5%, of the sample. The largest age group among participants is 20–29 years, making up 34.8% of the study population, followed by 30–39 years, representing 29.0% of the study population, and 40–49 years, forming 24.6% of the study population, while those above age 50 represented 11.6% of the study population. The most common level of experience is less than 10 years, representing 38.6% of the participant experience level, followed by participants who held 21–29 years of experience, which made up 32.9% of the experience level, and participants who had 11–20 years of experience, demonstrating 28.5% of the experience level. The distribution of experience levels has a *p*-value of 0.2, indicating no statistically significant difference among the groups. About 53.1% of the participants hold a bachelor’s degree in diagnostic radiography, followed by 18.4% of participants who hold a master’s degree and 10.6% of participants who hold a postgraduate certificate. The least common qualifications were represented by 6.8% of diplomas, 6.3% of postgraduate diplomas and 4.8% of doctoral degrees. Most participants have at least a degree-level education (*p* < 0.001).

### Analysis of patterns in knowledge

The analysis of the response to knowledge statements as shown in Table 1 reveals several significant patterns and implications for the training and education of diagnostic radiographers in dental radiography. Almost two-thirds of participants (75.4%) agreed or strongly agreed that they are aware of what dental radiography entails (*p* < 0.001). With respect to student training experience, responses were evenly distributed between those who have had experience with dental radiography during their student training (48.8% agreed or strongly agreed) and those who have not (45.9% disagreed or strongly disagreed) (*p* < 0.001).

**TABLE 1:** Knowledge of dental radiography.

Knowledge statements	Constructs	Strongly disagree		Disagree		Neutral		Agree		Strongly agree		Count	%
		Count	%	Count	%	Count	%	Count	%	Count	%		
I am aware of what dental radiography entails	K1	6	2.9	28	13.5	17	8.2	127	61.4	29	14.0	207	100.0
During my student training as a diagnostic radiographer, I have had experience with dental radiography	K2	23	11.1	72	34.8	11	5.3	76	36.7	25	12.1	207	100.0
I have gained dental radiography experience through work-integrated learning	K3	20	9.7	38	18.4	14	6.8	109	52.7	26	12.6	207	100.0
I have adequate knowledge on the necessary dental radiography projections	K4	39	18.8	102	49.3	23	11.1	28	13.5	15	7.2	207	100.0
I know the key anatomical positioning landmarks in dental radiography, like the bisecting angle technique for intraoral imaging and the Frankfort plane for panoramic imaging	K5	51	24.6	99	47.8	16	7.7	30	14.5	11	5.3	207	100.0

Note: Chi-square goodness of fit *p*-value < 0.001.

K, knowledge.

Sixty-five per cent of participants agreed or strongly agreed that they have gained dental radiography experience through work-integrated learning. However, a notable majority (68.1%) disagreed or strongly disagreed that they have adequate knowledge of the necessary dental radiography projections (*p* < 0.001). Furthermore, a significant portion of participants (72.4%) disagreed or strongly disagreed that they knew the key anatomical positioning landmarks in dental radiography, such as the bisecting angle technique for intraoral imaging and the Frankfort plane for panoramic imaging (*p* < 0.001).

### Analysis of patterns in attitudes

The analysis of responses to the attitudinal statements, as suggested in Table 2, provides insights into diagnostic radiographers’ attitudes towards dental radiography and their willingness to engage in related practices. Each statement has been assessed for the distribution of responses and the statistical significance of these distributions, as indicated by the Chi-square goodness of fit *p*-values. Below are the patterns observed:

**TABLE 2:** Attitudes to dental radiography.

Attitude statements	Constructs	Strongly disagree		Disagree		Neutral		Agree		Strongly agree		Count	%
		Count	%	Count	%	Count	%	Count	%	Count	%		
I am willing and keen to learn about dental radiography	A1	3	1.4	7	3.4	16	7.7	121	58.5	60	29.0	207	100.0
I’m open to gaining experience with dental radiography in a clinical setting	A2	3	1.4	6	2.9	12	5.8	118	57.0	68	32.9	207	100.0
Dental radiography deserves more recognition in diagnostic radiography	A3	2	1.0	5	2.4	20	9.7	101	48.8	79	38.2	207	100.0
I have a lot of interest in studying dental radiography	A4	6	2.9	13	6.3	14	6.8	82	39.6	92	44.4	207	100.0
Dental radiography is essential for diagnostic radiographers to study	A5	5	2.4	18	8.7	19	9.2	64	30.9	101	48.8	207	100.0

Note: Chi-square goodness of fit *p*-value < 0.001.

A, attitude.

About 87.5% of the participants agreed or strongly agreed that they are willing and keen to learn about dental radiography (*p* < 0.001). Most (89.9%) of the participants were open to gaining clinical experience in dental radiography (*p* < 0.001). A combined 87.0% of participants agreed or strongly agreed that dental radiography deserves more recognition within the field of diagnostic radiography (*p* < 0.001). A total of 84.0% of participants express a high interest in studying dental radiography (*p* < 0.001). A combined 79.7% of participants agreed or strongly agreed that dental radiography is essential for diagnostic radiographers to study (*p* < 0.001).

### Analysis of patterns in practice

The analysis of responses to the practice-related statements, as shown in Table 3, provides insights into the practical skills and self-assessed competencies of diagnostic radiographers in dental radiography. Each statement has been assessed for the distribution of responses and the statistical significance of these distributions, as indicated by the Chi-square goodness of fit *p*-values. The patterns observed and their implications are discussed below:

**TABLE 3:** Practice in dental radiography.

Practice statements	Constructs	Strongly disagree		Disagree		Neutral		Agree		Strongly agree		Count	%
		Count	%	Count	%	Count	%	Count	%	Count	%		
I can modify radiographic techniques for special needs clients, such as children or those with specific oral conditions	P1	16	7.7	79	38.2	27	13.0	75	36.2	10	4.8	207	100.0
I am well skilled in panoramic radiography, including client positioning, exposing, processing and critiquing for diagnosis	P2	18	8.7	55	26.6	24	11.6	92	44.4	18	8.7	207	100.0
I can analyse dental X-rays for dental material identification, cavities, gum disease and tooth root problems	P3	29	14.0	81	39.1	33	15.9	56	27.1	8	3.9	207	100.0
I am skilled in dental radiography techniques and able to practice confidently and independently	P4	42	20.3	100	48.3	19	9.2	35	16.9	11	5.3	207	100.0
I can use my knowledge of film-based dental radiographic techniques for digital imaging	P5	28	13.5	74	35.7	19	9.2	69	33.3	17	8.2	207	100.0

Note: Chi-square goodness of fit *p*-value < 0.001.

P, practice.

A proportion of participants (45.9%) disagreed or strongly disagreed that they can modify radiographic techniques for special needs clients, indicating a gap in this area (*p* < 0.001). Although 41% agreed or strongly agreed, the near-equal split suggests that this is an area requiring focused training to ensure all radiographers can confidently handle special needs clients (*p* < 0.001). A combined 53.1% of participants agreed or strongly agreed that they are well skilled in panoramic radiography, while 35.3% disagreed or strongly disagreed (*p* < 0.001).

Many participants (53.1%) disagreed or strongly disagreed with their ability to analyse dental images, indicating a substantial gap in analytical skills (*p* < 0.001). Only 31.0% agreed or strongly agreed with this statement (*p* < 0.001). Most participants (68.6%) disagreed or strongly disagreed with the statement that they were skilled in dental radiography techniques and able to practice dental radiography techniques confidently and independently (*p* < 0.001). The low percentage of agreement (22.2%) suggests that substantial efforts are needed to build confidence and proficiency through practical training, mentorship and continuous professional development (*p* < 0.001). A portion of participants (49.2%) disagreed or strongly disagreed that they can apply their knowledge of film-based techniques to digital imaging (*p* < 0.001). Although 41.5% agreed or strongly agreed, this nearly even split highlights the necessity for bridging the gap between traditional film-based techniques and modern digital imaging (*p* < 0.001).

### Correlation and structural relationship analysis

To explore the interrelationships between knowledge, attitudes and practices in dental radiography, both bivariate and multivariate statistical techniques were applied. This dual approach was used to validate the study’s correlational design and provide robust evidence of the nature and strength of associations among the key constructs.

### Pearson correlation analysis

Pearson’s correlation coefficients were computed to test the linear relationships between the three core constructs: Knowledge (K), Attitudes (A) and Practices (P). The results are summarised in Table 4.

**TABLE 4:** Pearson correlation analysis.

Variables	Pearson correlation (r)	Sig. (two-tailed)	Interpretation
Knowledge and practice	0.644	< 0.001	*Strong positive, significant correlation*
Knowledge and attitude	0.067	0.34	Weak, *not significant* correlation
Attitude and practice	0.155	0.026	Weak *but statistically significant* correlation

A strong and statistically significant positive correlation was observed between Knowledge and Practice (*r* = 0.644, *p* < 0.001), indicating that participants with higher knowledge levels demonstrated better practical competencies in dental radiography. A weak, non-significant correlation was found between Knowledge and Attitude (*r* = 0.067, *p* = 0.340), suggesting that knowledge may not directly influence attitudinal dispositions. A weak but statistically significant positive correlation was recorded between Attitude and Practice (*r* = 0.155, *p* = 0.026), implying a marginal association between positive attitudes and improved practice. These results confirm that the study design aligns with a correlational framework and supports the hypothesised relationships among the constructs.

## Discussion

The gender disparity trend suggests a higher representation of females in the diagnostic radiography profession within the sample population. The higher proportion of younger participants within the diagnostic radiography field reflects recent graduates or early-career professionals. The sample also suggested a diverse range of experience levels among participants without a dominant trend and a high level of formal education among diagnostic radiographers in the sample, with a notable proportion pursuing advanced qualifications.

The study findings indicated that the majority of participants had a significant gap in technical-specific knowledge and dental positioning landmarks (Table 1). Thus, the overall patterns observed suggest the need for targeted educational interventions to enhance specific technical knowledge and standardise training experiences. The current study findings are in agreement with several authors, such as McNulty et al. (2021), Van de Venter and Engel-Hills (2022) and Hudson et al. (2022), whose studies have alluded to radiography training curriculum to be more responsive, structured and focused, as this would equip graduates with expert knowledge and enable them to navigate the complexities of the modern healthcare requirements. The study findings are also aligned with the recommendations made by Chen et al. (2022) and Mahasneh et al. (2022), who highlight the need for greater emphasis on developing innovative dental radiography courses for diagnostic radiography students. A study conducted by Mahabob et al. (2021) on dental professionals’ knowledge of dental radiation protection found that improving technical knowledge in dental imaging reduced repeat acquisition and allowed for improved radiation safety and optimised image quality. Likewise, a study conducted in India encouraged the need for more structured dental radiography training programmes, for health professionals, as including technical specific knowledge and knowledge on advanced dental imaging modalities such as the cone beam computed tomography (CBCT) would allow the dental professionals to select the best dental imaging modality that would provide maximum diagnostic benefits for patients (Rai et al. 2018; Thakkar et al. 2019). This points to the need to develop focused, structured dental radiography training programmes not just for dental professionals but for all health professionals involved in dental imaging. Hendricks, Hartman and Olckers (2021) reiterated the need for interprofessional teamwork and collaborative efforts when work is of an interactive nature among health professionals. This method of teamwork pedagogies should be considered for alignment of the practice and training of dental radiography across these disciplines.

Additionally, results were evenly distributed between participants who agreed and disagreed with having had experience in dental radiography during their student training (Table 1). This variability suggests differences in curriculum or clinical training exposure across institutions. It can be assumed that if dental radiography is within the SA scope of practice for diagnostic radiographers, then it would be covered in the undergraduate curriculum. However, given the paucity of available literature in this field, it is difficult to verify this without an examination of the curriculum. Given that this article forms part of a larger study, some information will be reported elsewhere. The findings from this study, however, allude to the need for standardising dental radiography training during student education, as this could help bridge this gap and ensure consistent learning experiences. These findings are similar to those reported by McNulty et al. (2021) and England et al. (2017), who have shown that the radiography training curriculum differed across Europe. These authors have suggested that the main reason for a difference in the training curriculum was because of student exposure to imaging modalities and the duration of clinical time spent within these modality-specific areas (McNulty et al. 2021 and England et al. 2017). A study conducted in Nigeria demonstrated this notion, where students’ practical training in dental radiography differed as a result of limited or no dental equipment (Akanpiwo et al. 2018). Likewise, in Jordan, a poor understanding of quality assurance for intraoral dental equipment was cited as a result of poor to no clinical exposure of diagnostic radiographers to intraoral dental equipment (Mahasneh et al. 2022). There is a need for further research in this area to examine the availability of dental resources in industry to support student training in dental radiography. This also calls for a review by regulators, academics and industry to review the extent to which dental radiography is covered in undergraduate curricula for diagnostic radiographers in SA.

The study findings also suggest a positive and receptive attitude towards dental radiography among study participants, where the majority of the participants showed a high level of willingness and enthusiasm among diagnostic radiographers to learn about dental radiography, indicating strong support for educational initiatives (Table 2). Most participants appeared to be open to gaining practical experience, underscoring the importance of incorporating clinical training opportunities in dental radiography programmes. This reflects a strong interest in studying dental radiography, which can be leveraged to develop and promote specialised educational programmes. Additionally, most participants felt that dental radiography needs more recognition in radiography. In contrast, a study conducted by Manning-Stanley and Kirby (2022) ranked dental radiography as the lowest preference for selection as a specialised study according to the undergraduate diagnostic radiography students in the UK. As indicated, there is limited published evidence on diagnostic radiographers’ practice in dental radiography in South Africa. Additionally, the current curricula on undergraduate training in radiography were not publicly available. This study aimed to obtain baseline information related to radiographers’ knowledge, attitudes and practices related to dental radiography. This will be listed as a limitation of the study. However, the findings of this study point to the need to provide a strong foundation for enhancing training programmes and to promote a possible specialisation in the field of dental radiography.

The participants also indicated that they were not confident to practice dental radiography techniques independently, thus suggesting a gap in practical skills (Table 3). Furthermore, a notable gap was reported in applying film-based knowledge to digital imaging, underscoring the need for focused training on modern techniques. This implies that practical skills and confidence need to be addressed through targeted training and professional development initiatives. This approach will ensure diagnostic radiographers are well equipped to provide high-quality care and stay current with advancements in dental radiography. These findings concur with several studies that have reviewed dental professionals’ need to be more skilled in advanced dental radiography modalities, as alluded to above in modalities such as the CBCT and digital dental imaging. In these studies, enhancement of practical skills was seen as an effective means to develop broad skill sets and to operate dental equipment more effectively (Rabhat et al. 2011; Rai et al. 2018; Thakkar et al. 2019). This points to the need for a constant review of training programmes to ensure that professionals have the required skills to practice in dental radiography. The findings of this study suggest that there could be significant gaps in participant practical skills too and hence the need for further research and review of dental training programmes for diagnostic radiographers in South Africa.

Furthermore, participants had limited ability to analyse dental images, indicating a substantial gap in analytical skills. This deficiency in the ability to analyse dental images highlights the need for enhanced training in radiographic interpretation. The findings of this study thus relate to research studies in South Africa that also highlight the benefits of increasing radiographers’ analytical skills in radiographic image analysis and interpretation. Budhu et al. (2022) iterate that, unlike in other countries, image analysis and interpretation do not form part of the SA diagnostic radiographers’ scope of practice. Van der Venter and Ham-Baloyi (2019) further add that under the current scope of practice, radiographers are only permitted to comment on the examination performed and not provide a diagnosis. This limitation in image diagnostics and interpretation from a SA context contrasts with studies conducted in other countries that have highlighted the need to develop focused image analysis and interpretation courses for radiographers (Budhu et al. 2022; Hardy et al. 2016). These studies have shown that equipping radiographers with such analytical skills and knowledge could yield positive outcomes such as improvements in patients’ waiting times, early disease detection and better patient management (Bwanga, Sichone & Kafwimbi 2020; Hardy et al. 2016).

### Study limitations

Despite the valuable insights, the study could have incorporated focus group discussions and in-depth interviews to provide additional data that could enhance understanding in this area to further enhance the quantitative data. This study was also limited by the paucity of available data on the current diagnostic radiography curriculum. The targeted sample size was *n* = 358; however, the study obtained a response rate of 57.8% (*N* = 207). Some of the possible reasons for the low response rate were attributed by group administrators to some respondents’ indication that they were not interested or they did not have time to complete the survey, and others chose not to participate because of a lack of interest or perceived relevance. Despite these limitations, the study does provide pertinent data to use as a baseline for further research studies in this field.

### Recommendations

As dental radiography features across multiple disciplines, it may be of interest to compare all health professionals’ studies of a similar nature and to include more provinces in SA. It is recommended that the targeted educational interventions, such as continuous professional development (CPD) activities, be encouraged to enhance dental-specific knowledge and practical skills for diagnostic radiographers in SA.

### Contributions to research

This study reiterates the value of investing in continuous professional development activities to address identified knowledge and skill gaps among diagnostic radiographers. Such activities will provide the professionals the time and space to build on existing knowledge and skills. Additionally, the study highlights the need for an evidence base to ensure that such activities are responsive to the specific needs of diagnostic radiographers and are grounded in the real-world settings.

## Conclusion

There were noted inconsistencies in diagnostic radiographers’ dental-specific knowledge and practical skills in dental radiography, thus reiterating the importance of investing in continuous professional development activities for diagnostic radiographers in dental radiography. The study findings further suggested that participants had exposure to dental radiography in the undergraduate training, but that there was variability in how this training was offered. In general, there was a positive attitude among participants who showed a willingness to learn dental radiography. Overall, the study findings highlight the need for further research to explore and understand the possible inconsistencies in undergraduate exposure to dental radiography to better prepare the graduate for the health system.
